# Synthesis of Methacryloylated Hydroxyethylcellulose and Development of Mucoadhesive Wafers for Buccal Drug Delivery

**DOI:** 10.3390/polym15010093

**Published:** 2022-12-26

**Authors:** Fhataheya Buang, Afroditi Chatzifragkou, Mohd Cairul Iqbal Mohd Amin, Vitaliy V. Khutoryanskiy

**Affiliations:** 1 Reading School of Pharmacy, University of Reading, Whiteknights, Reading RG6 6AD, UK; 2 Centre for Drug Delivery Technology, Faculty of Pharmacy, Universiti Kebangsaan Malaysia, Jalan Raja Muda Abdul Aziz, Kuala Lumpur 50300, Malaysia; 3 Department of Food and Nutritional Sciences, University of Reading, Whiteknights, Reading RG6 6AD, UK

**Keywords:** hydroxyethylcellulose, mucoadhesion, methacryloyl, transmucosal delivery, wafers

## Abstract

Non-ionic hydroxyethylcellulose (HEC) has limited mucoadhesive properties for application in transmucosal drug delivery. In this study, HEC was chemically modified by reaction with glycidyl methacrylate. This allowed introducing the methacryloyl groups to HEC structure to make it capable of forming covalent bonds with the sulfhydryl groups present in the mucin glycoprotein to achieve enhanced mucoadhesive properties. The results showed a successful modification of HEC as confirmed by ^1^H NMR and FTIR spectroscopies. The quantification of methacryloyl moieties was conducted using HPLC. The toxicity studies using in vivo planaria acute toxicity assay, in vivo planaria fluorescent test, and in vitro MTT assay with Caco-2 cell line confirmed that the chemical modification of HEC does not result in any toxicological effects. Mucoadhesive wafers were developed based on parent and modified HEC as a model dosage form for buccal delivery. The mucoadhesive properties of modified HEC assessed using a tensile test were found to be significantly better compared to unmodified HEC.

## 1. Introduction

The delivery of drugs through mucosal membranes lining the body is a non-invasive option for achieving local and systemic effects. Transmucosal drug delivery offers advantages such as increased drug residence time, improved bioavailability, and avoidance of the first-pass effect or pre-systemic metabolism [1,2,3]. Oromucosal, gastrointestinal, ocular, vaginal, intravesical, nasal, and rectal routes are among the established routes of transmucosal drug delivery. In any of the mentioned routes, poor drug retention on the site of action is usually an issue. Thus, to increase the drug residence on the mucosa, mucoadhesive materials are commonly used in the formulations as they facilitate dosage form adhesion to the tissues [4].

Cellulose and its derivatives are biocompatible, renewable, and non-toxic polysaccharides. They belong to the first generation of mucoadhesive polymers as they may interact with mucosal surfaces via physical attraction forces such as hydrogen bonding [5,6]. Compared to cationic and anionic polymers, the non-ionic hydroxyethylcellulose (HEC) exhibits limited mucoadhesive characteristics [7,8].

Blending HEC with other mucoadhesive polymers is one of the methods used for enhancing mucoadhesive properties of dosage forms. However, the dosage form’s adhesiveness may potentially be impacted by the interpolymer complexation between HEC and other polymers [9]. Therefore, alternative strategy to enhance mucoadhesive properties of HEC is through its chemical modification to introduce adhesive groups. For example, modification of HEC with cationic and thiol groups has been reported previously by other researchers [10,11]. Previously we demonstrated that introduction of methacryloyl groups into chitosan [12], gellan gum [13] and poly(2-ethyl-2-oxazoline) [14] leads to a substantial enhancement in the mucoadhesive properties of these polymers. This modification was achieved by reacting chitosan, gellan gum and ethylene imine-*co*-2-ethyl-2-oxazoline with methacrylic anhydride. The enhancement in mucoadhesive properties is due to the ability of methacryloyl groups to form covalent bonds with thiol groups present in mucin under physiological conditions.

In this study, we have modified non-ionic HEC by reaction with glycidyl methacrylate as a new strategy to introduce mucoadhesion-enhancing groups into polymers. The resulting derivatives were characterized using ^1^H NMR and FTIR spectroscopies as well as hydrolysis with subsequent quantification of methacrylic acid with HPLC. The toxicological properties of these new HEC derivatives were evaluated using acute toxicity and fluorescence assays in planaria as well as MTT cytotoxicity assay in Caco-2 cells. The parent as well as the modified polymers were subsequently formulated into the wafers as a model dosage form for buccal drug delivery. The porosity, mechanical and mucoadhesive properties of these wafers were evaluated.

## 2. Materials and Methods

### 2.1. Materials

HEC (720 kDa), triethylamine (TEA), tributyl ammonium bromide (TAB), glycidyl methacrylate (GMA), hydrochloric acid, benzalkonium chloride, sulfuric acid, methacrylic acid and sodium hydroxide were purchased from Sigma Aldrich Co., Ltd., Gillingham, UK. N,N-dimethylformamide (DMF) was supplied by SLS Supplies Ltd., Nottingham, UK.

Cell culture materials DMEM High Glucose (Capricorn Scientific GMbH, Germany), foetal calf serum (GE Healthcare Life Sciences, Chicago, IL, USA), penicillin/streptomycin (Nacalai Tesque Inc., Kyoto, Japan), CellTiter 96 Aqueous MTS reagent powder (Promega Corporation, Wisconsin, USA) were used for cell viability assay. The Caco-2 cells were received from Dr. Sharifah Aminah, Faculty of Pharmacy, in UiTM Puncak Alam, Malaysia.

The freshly excised sheep upper and lower lips were sourced from PC Turner Abattoir (Farnborough, Hampshire, UK).

### 2.2. Modification of HEC

1% solution of HEC (*w*/*v*) was prepared by dissolving HEC in 0.1 M NaOH. Then TEA was added to HEC solution as a catalyst. GMA was added to these solution mixtures at different molar ratios, as shown in Table 1, and constantly stirred at 25 °C for 24 h. The reaction products were purified using a dialysis via membranes with molecular weight cutoff of 12–14 kDa. Deionised water was changed 8 times (4.5 L) a day for over 48 h during dialysis. The final products were subsequently freeze-dried.

### 2.3. H Nuclear Magnetic Resonance Spectroscopy (^1^H NMR)

Polymer solutions (20 mg/mL) were prepared in D_2_O in NMR tubes of 5 mm diameter. The ^1^H NMR spectra were recorded using a 400 MHz Ultrashield Plus^TM^ B-ACS 60 spectrometer (Bruker UK Ltd., Coventry, UK) and were analysed using MestReNova (Mnova) Version 6.0.2-5475.

### 2.4. Fourier Transform Infrared (FTIR) Spectroscopy

FTIR spectra of freeze-dried samples were recorded using Spectrum 100 FTIR Spectrophotometer (Perkin–Elmer UK Ltd., Buckinghampshire, UK) with scanning from 4000 to 650 cm^−1^ at 4 cm^−1^ resolution, and accumulation of 16 scans. The data were analysed using a six-scan average per sample generated by Spectrum One software.

### 2.5. High-Performance Liquid Chromatography (HPLC)

For the analysis of methacryloyl groups content, 40 mg of polymer samples were dissolved in 8 mL of 0.01 M sulfuric acid and solutions were refluxed for 4 h at 50 °C. Methacrylic acid formed as a result of this reaction was quantified using HPLC.

The HPLC procedure for the analysis of methacrylic acid was adapted from Paleologos and Kontaminas [15], and was carried out on an Agilent Infinity 1200 HPLC system with an Aminex 87H (Biorad, Watford, UK) column at 40 °C. Isocratic elution was applied at 0.6 mL·min^−1^ with 0.01 M sulfuric acid solution and methacrylic acid detection was performed in a diode array detector (Agilent Infinity 1200 Series, Didcot, UK) at 200 nm wavelength.

Methacrylic acid was dissolved in 0.01 M sulfuric acid to form the standard stock solution, which was diluted with deionised water to form standard solutions with concentrations ranging from 0.1 to 59.0 µmol/mL, used for the generation of external calibration curve and methacrylic acid quantification in the samples.

### 2.6. Planarian Acute Toxicity Assay

*Schmidtea mediterranea* planaria were provided by Oxford Brookes University and were kept in artificial pond water (APW: 5 M NaCl, 1 M CaCl_2_, 1 M MgSO_4_, 1 M MgCl_2_ and 1 M KCl) at room temperature. Planaria were given chicken liver once a week, and the APW was changed every week following their feeding. Planaria (1.0–1.5 cm long) were placed each in 24 wells of a plate culture using a slightly modified version of the procedure [16,17]. Briefly, 1 mL of HEC and HECGMA solutions at various concentrations (0.05% *w*/*v*, 0.10% *w*/*v*, 0.25% *w*/*v*, 0.50% *w*/*v* and 1.00% *w*/*v*) were added into each well. Solution of 1% *w*/*v* benzalkonium chloride (BAC) in APW was used as a positive control that typically causes severe irritation of mucosal membranes [18]. All test materials were dissolved in APW. The plates were stored at room temperature in the dark. The number of living and dead planaria was determined after 24, 48, and 72 h of the acute toxicity test. Planaria that did not move after a gentle agitation were considered dead.

### 2.7. Planarian Toxicity Fluorescent Assay

Following the experiments on acute toxicity assay, where the worms were exposed to 1.0% *w*/*v* polymer solutions for 24 h, these planaria were subsequently exposed to 0.1% *w*/*v* sodium fluorescein solution in APW for 1 min. The worms were then washed in APW for 15 min to remove residual dye. In order to immobilise the planaria, a glass slide containing the worms was covered with a few drops of a 2.0% *w*/*v* agarose solution and placed on a flat surface of ice flakes (−0.5 to −0.8 °C) until the gel solidified. Leica MZ10F stereomicroscope (Leica Microsystems Ltd., Wholesaler, UK) equipped with DFC3000G digital camera at 2.0× magnification, 160 ms exposure duration, and gamma 0.7 were used to record fluorescence images of the worms. Permeation of sodium fluorescein into the worms was evaluated using ImageJ software (version 1.8.0_112) as described in Shah et al. [16]. The acquired mean value was normalised by dividing the fluorescence intensity by the total area (in cm^2^) of each planaria.

### 2.8. In Vitro Cytotoxicity of Polymers

The cytotoxicity of each polymer was evaluated using Caco-2 cells. The cells were grown in DMEM High Glucose fortified with 10% foetal calf serum and 1% penicillin/streptomycin. It was kept at 37 °C in an incubator with 5% CO_2_ and 100% relative humidity.

At a density of 1 × 10^4^ cells per well, cells were seeded in 96-well plates and incubated for an overnight period at 37 °C in humidified air containing 5% CO_2_ to promote cell attachment. The cells were then treated with various concentrations of the polymers (1%, 0.5%, 0.25%, 0.1% and 0.05% *w*/*v*) for 24 h. The negative control group consisted of untreated cells and was considered as 100% of viable cells. The media were changed with fresh growth medium following the end of every treatment. Each well received 20 μL of 5 mg/mL MTT solution (in the dark). The cells were further incubated for 4 h at 37 °C in a humidified 5% CO_2_ incubator. 100 μL of DMSO was added, mixed thoroughly and incubated for 10 min. The absorbance was measured at 540 nm with Infinite 200 PRO microplate reader (Tecan Group Ltd., Maennedorf, Switzerland).

### 2.9. Preparation of Wafers

The wafers were prepared from 1% *w*/*v* solutions of HEC and its derivatives in deionised water. 1.5 g of HEC, and HECGMA solutions were poured into each well in 24 well plates. The plate was covered with holed aluminium foil and was left under a fume hood for an hour. It was then frozen in a freezer at −20 °C overnight. The wafers were prepared by freeze-drying in a Heto Power Dry LL3000 Freeze Dryer (Thermo Scientific UK Ltd., Leicestershire, UK) over 48 h. The wafers were placed in sealed containers and stored in a fridge at 4 °C.

### 2.10. Physical Characterisation of Wafers

Wafers were examined for physical features (colour and texture). A digital microbalance was used to weigh the wafers, and their average weight ± standard deviations were calculated. The wafers were each measured for thickness using an electronic Vernier calliper, and the average thickness ± standard deviations were calculated. SEM analysis of the wafers provided more information on their porous structure. The wafers were mounted on an aluminium stud and secured with double-sided carbon tape adhesive. SEM images were generated using FEI Quanta 600 FEG.

### 2.11. Ex Vivo Mucoadhesion Study of Wafers

The method was slightly modified from several studies [19,20,21]. A TA-XT Plus Texture Analyser (Stable Micro Systems Ltd., Surrey, UK) with a 5 kg load cell was used to study the mucoadhesive properties of all the formulations. Sheep buccal tissue was cut into squares and secured onto mucoadhesion rig with 20 mm opening. Upon testing, the device and tissues were immersed in a 37 °C water bath.

The wafers were attached to the 12 mm diameter aluminium probe with sticky adhesive tape and lowered to contact the mucosa. The following test parameters were used: pre-speed test 0.5 mm/s; test speed 0.5 mm/s; post-speed test 1.0 mm/s; applied force 0.5 N; contact time 60 s; trigger type auto; trigger force auto; and return distance 20 mm.

### 2.12. Statistical Analysis

SPSS (version 17) was used to perform a two-tailed Student *t*-test as a statistical tool with *p* values < 0.05 considered statistically significant.

## 3. Results and Discussion

### 3.1. Synthesis of Methacryloylated HEC

**Figure 1:**
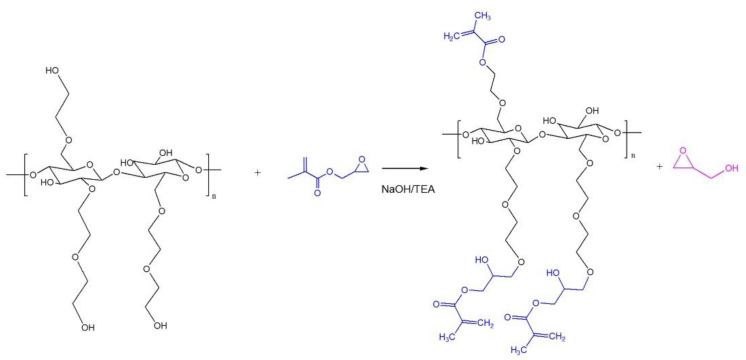
Proposed reaction scheme of HEC with glycidyl methacrylate at alkaline conditions (pH 13.3).

**Figure 2:**
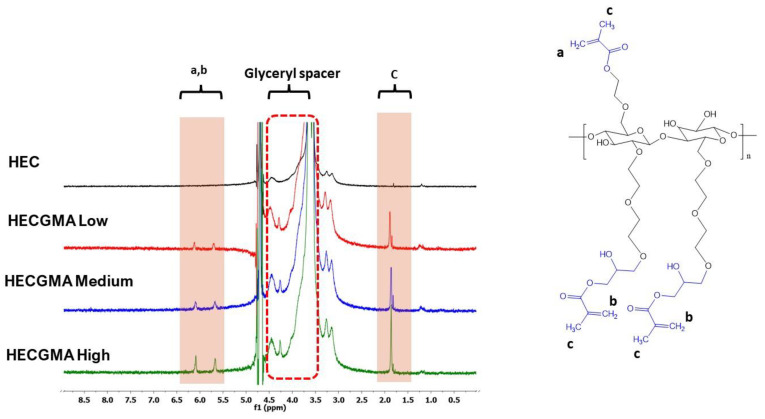
Structure and ^1^H NMR spectra of unmodified HEC and HECGMA prepared at various molar ratios of HEC to GMA.

It can be expected that the reaction of GMA with HEC leads to formation of methacryloylated derivatives (Figure 1), which is similar to the reactions of this reagent with other hydroxyl-containing polymers reported in the literature [22,23]. In general there are two reaction routes possible with the use of GMA in chemical modification, via transesterification and epoxide ring opening mechanisms [22,23,24,25,26]. We conducted the synthesis in alkaline protic solvent (containing NaOH and TEA as bases) which resulted in the reaction favouring epoxide ring opening than transesterification as reported by Fajardo et al. and Reis et al. [22,27]. The structure of the resulting derivatives of HEC was evaluated using ^1^H NMR spectroscopy (Figure 2).

The ^1^H NMR spectra of modified HEC show the signals at 5.69 and 6.10 ppm, which correspond to the protons of methacryloyl groups [12,13]. The signals that appeared in the spectra of methacryloylated HEC at 1.89 ppm correspond to protons of methyl groups from methacryloylation [13]. The peaks at 1.22 and 1.82 ppm belong to unidentified structure within HEC, which was similarly found and reported by Ray et al. [28].

Unfortunately, the extent of HEC methacryloylation cannot be evaluated accurately using the analysis of ^1^H NMR spectra. HEC has a complex structure similarly to other heteropolysaccharides that generates broad signals in the ^1^H NMR spectra, which overlap with glyceryl spacer (4.50–3.50 ppm) in methacryloylated derivatives [22,23].

**Figure 3:**
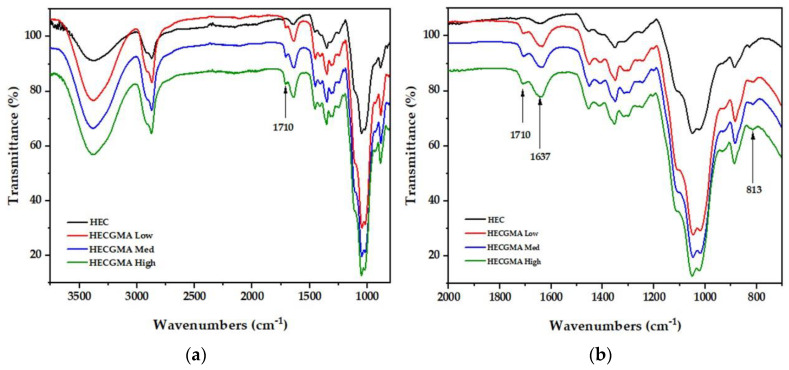
FT-IR spectra of unmodified HEC and HEC modified with GMA at a low, medium, and high molar ratio with wavenumbers (**a**) at the range of 3750–750 cm^−1^; and (**b**) at the range of 2000–750 cm^−1.^.

Figure 3 shows the infrared spectra for unmodified HEC and HECGMA. The successful modification of HEC with GMA was confirmed by the introduction of a new absorbance band at 1710 cm^−1^ in the HECGMA High spectrum attributed to the stretching frequency of C=O, while absorbance band at 1637 cm^−1^ is due to C=C groups [25]. In Figure 3b, the band 813 cm^−1^ is the characteristic of CH out-of-plane vibration present in all HECGMA [24,25]. This band results from the presence of methyls of methacrylol groups. It was observed that all the above bands mentioned were present following modification of HEC at a high molar ratio to GMA.

Quantification of methacrylic acid recovered from hydrolysed modified HEC samples showed that the methacryloyl groups content in HECGMA High, Medium and Low were 173.50 ± 32.84 µmol/g, 72.43 ± 6.16 µmol/g and 64.49 ± 5.98 µmol/g, respectively. The results for negative control (unmodified HEC) show no presence of methacryloyl groups (data are shown in Supplementary Information: Figure S1 and Tables S1 and S2).

### 3.2. Acute Toxicity Assay and Fluorescent Assay in Planaria

Toxicology screening of the HECGMA was performed using fixed-dose procedures on planaria worms. Planaria were used in toxicology screening of chemicals because of their permeable epithelia that may absorb low molecular weight compounds from their environment [29]. The acute toxicity assay using planaria revealed that HECGMA derivatives at the studied concentrations (0.01% *w*/*v*, 0.05% *w*/*v*, 0.25% *w*/*v*, 0.50% *w*/*v* and 1.00% *w*/*v*) do not cause death in planaria for 24 h, 48 h and 72 h of exposure. The exception is the control group of worms exposed to 1% BAC, which resulted in dead planaria, with no signs of worm movement at all.

**Figure 4:**
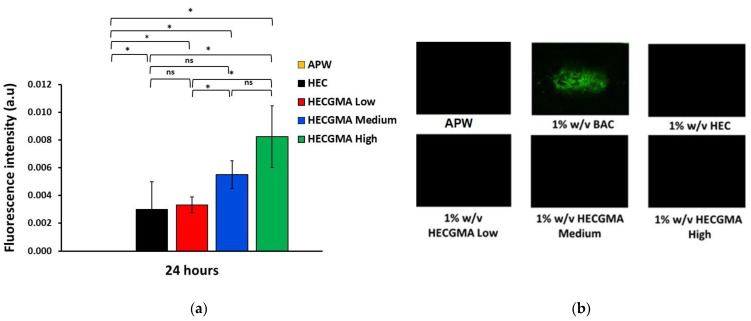
Fluorescent assay using planaria. (**a**) Histograms representing the relative intensity of fluorescence in planaria after the exposure to 1 % *w*/*v* of unmodified HEC, HECGMA Low, HECGMA Medium and HECGMA High. (**b**) Images of planaria worms after exposure to APW, 1% *w*/*v* of BAC, HEC, HECGMA Low, HECGMA Medium and 1% HECGMA High. Data show the mean ± SE (n = 3). * Statistically significant according to *t*-test; *p* < 0.05, ns = not significant.

Fluorescent assay was previously developed by our research group using planaria model to evaluate the effect of irritant chemicals on the permeability of their epithelial membranes [16]. The assay is based on disruption of planaria epithelia caused by irritant chemicals. When planaria are exposed to an irritant chemical the integrity of their epithelium is disrupted and this facilitates penetration of fluorescein sodium into their body. This is evaluated through the analysis of fluorescent microphotographs of worms following their exposure first to a chemical of interest, then to solution of sodium fluorescein. Fluorescent assay was carried out to evaluate the effect of 1% *w*/*v* HEC and HECGMA on planaria epithelia during 24 h exposure. Figure 4 presents some exemplar fluorescence images as well as the results of image analysis after 24 h expressed as fluorescence intensity values. A 24 h exposure of planaria to different polymers indicated that even unmodified HEC causes a statistically significant enhancement (*p* < 0.05) of fluorescein penetration into the worms’ body compared to the negative control with artificial pond water (APW). It is well known that HEC is widely used in various topical and mucosal formulations and it is a biocompatible and non-irritant polymer at this concentration [30]. Exposure of planaria to HECGMA Low and Medium did not cause a significant increase in the fluorescence intensity compared to unmodified HEC (*p* > 0.05); this indicates that these two derivatives have non-irritant properties like HEC. However, exposure of planaria to HECGMA High resulted in a 2× times greater fluorescence intensity compared to unmodified HEC, which indicates that this sample is potentially more irritant.

### 3.3. In Vitro Cytotoxicity

**Figure 5:**
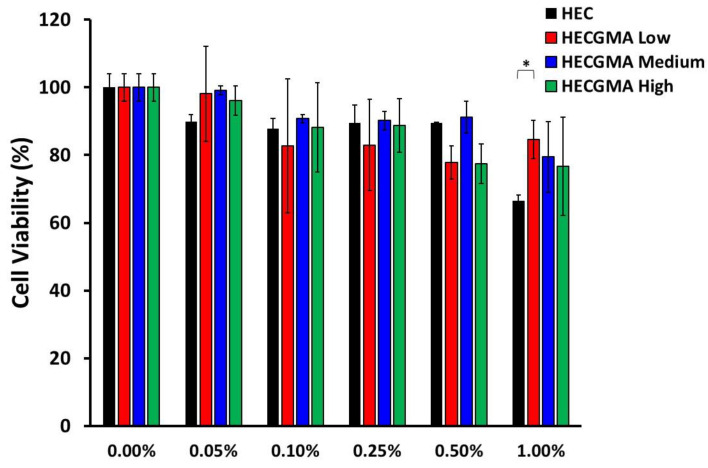
Cell viability evaluated using MTT assay with the percentage of viable cells after the exposure to: 0%, 0.05%, 0.10%, 0.25%, 0.50%, and 1.00% of HEC and HECGMA derivatives at 24 h. Data show the mean values ± SD (n = 3). * Statistical significance is shown according to *t*-test; *p* < 0.05.

The cytotoxicity of HEC and HECGMA derivatives was studied using the Caco-2 cell line in a concentration range of 0.05 to 1% *w*/*v*. MTT results showed that the cell viabilities are comparable for HEC and all HECGMA derivatives and all are above 60% after 24 h (Figure 5). In the majority of cases, the difference between the unmodified HEC and HECGMA derivatives was not statistically significant (*p* > 0.05), which indicates that chemical modification of HEC with methacryloyl groups does not cause an increase in the polymer toxicity.

### 3.4. Preparation and Physical Characterisation of Wafers

**Figure 6:**
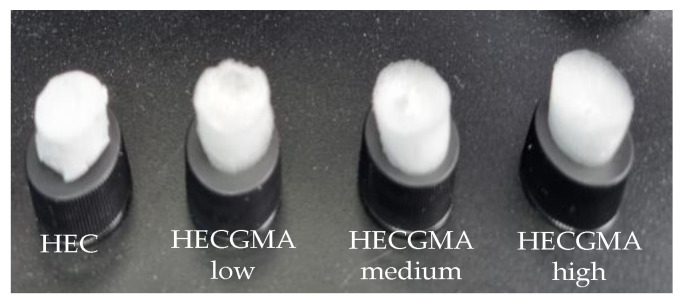
Physical appearance of lyophilised wafers based on HEC and HECGMA derivatives.

Lyophilized formulations, containing water-soluble polymers, often form wafers that are widely reported in the literature for application in buccal drug delivery. In the present work, the unmodified HEC and new HECGMA derivatives were used to prepare wafers as model dosage forms. The wafers developed in our work were light, spongy and white with a soft and smooth texture. The texture of wafers is important as it influences the oral intake of medicine. Grittiness from the product formulations gives an unpleasant mouthfeel after intake [31]. All the formulations were easily removed from the mould. Selected images of these wafers are shown in Figure 6.

**Figure 7:**
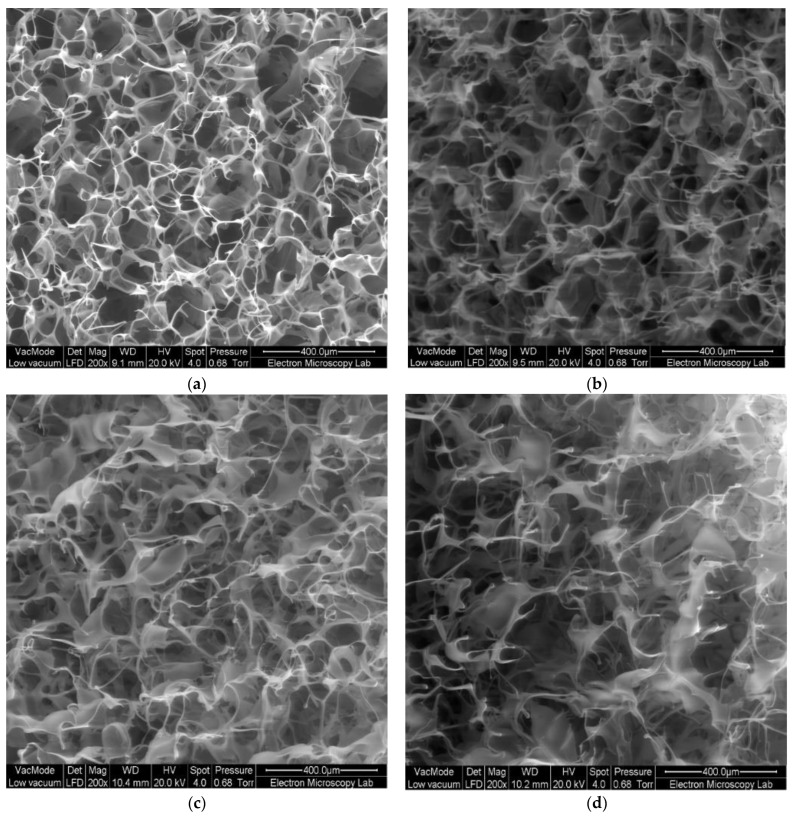
SEM images of lyophilised wafers of (**a**) unmodified HEC, (**b**) HECGMA Low, (**c**) HECGMA Medium and (**d**) HECGMA High.

The average diameter of these wafers was 12 mm. The morphology of the wafers was examined using SEM (Figure 7). The porosity of wafers was conferred by freeze-drying as a result of the elimination of ice crystals via the sublimation process [31].

### 3.5. Ex Vivo Evaluation of Mucoadhesive Properties of Wafers

**Figure 8:**
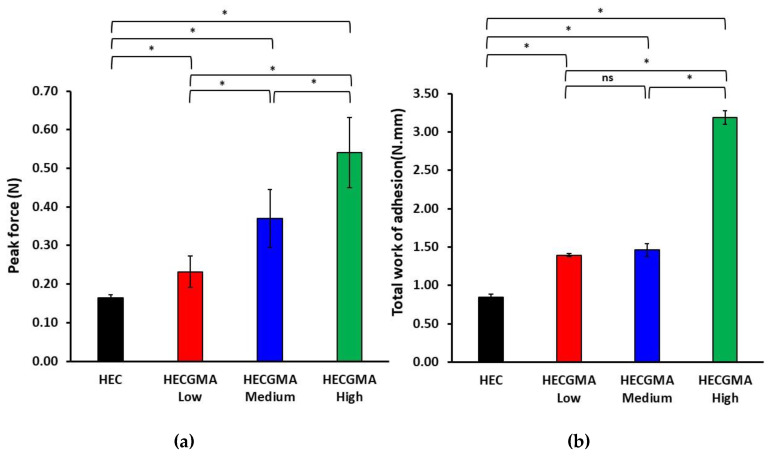
The results of tensile test to evaluate mucoadhesive properties of the wafers based on unmodified HEC and HECGMA derivatives: (**a**) Peak force (N) and (**b**) Total work of adhesion (mm·N). Data show the mean values ± SD (n = 5). * Statistically significant according to *t*-test; *p* < 0.05; ns = not significant.

Adhesion of the wafers to freshly excised sheep buccal mucosa was evaluated using a tensile test, established in the literature on mucoadhesive dosage forms [1]. This test provides two main parameters such as the peak force or maximal detachment force and the total work of adhesion, calculated as the area under the detachment curve. Figure 8 shows the results of the tensile test evaluating mucoadhesive properties of the wafers, including the data on the peak force and the total work of adhesion. As expected, the wafers prepared from unmodified HEC exhibited relatively modest adhesion because of the non-ionic nature of this polymer [1]. However, a statistically significant improvement in adhesive properties was observed for the wafers prepared from HECGMA derivatives. The adhesive properties generally improve for the derivatives with greater content of methacryloyl groups in the polymer. HECGMA High derivative exhibited the greatest mucoadhesive performance, whose peak force and the total work of adhesion were 3.27× and 3.79× greater compared to these parameters recorded for the wafers composed of unmodified HEC.

Thus, methacryloylated HEC exhibits enhanced mucoadhesive properties and can be used to formulate dosage forms for buccal drug delivery. The advantage of methacryloylated HEC compared to other mucoadhesive polymers commonly used for buccal delivery such as chitosan [32], sodium carboxymethylcellulose, poly(acrylic acid) derivatives and carragennan [33], pectin [34] and alginates [35] is its non-ionic nature. Non-ionic polymers have better compatibility with ionic drugs as they will not form insoluble complexes that may affect release characteristics.

## 4. Conclusions

The present study demonstrated that poor mucoadhesive properties of HEC could be significantly improved by introduction of methacryloyl groups into the structure of this non-ionic polymer. This was achieved by reaction of HEC with glycidol methacrylate. The structure of resulting HEC derivatives was confirmed using FTIR and ^1^H NMR spectroscopies as well as by HPLC-based assay to quantify the presence of methacrylic acid in the hydrolysed polymers. The tests performed using planaria and Caco-2 cells indicated that the new HEC derivatives do not show any adverse toxicological reactions similarly to unmodified HEC. All these polymers were then prepared as wafers and their mucoadhesive properties were evaluated using a tensile test in freshly excised sheep buccal mucosal model. All HEC derivatives exhibited superior mucoadhesive properties compared to unmodified HEC and the greater presence of methacryloyl groups improved adhesiveness to mucosa. The new excipients based on HECGMA can be easily synthesized and have solubility in water. Potentially these polymers can be used not only for the preparation of wafers for buccal drug delivery but also for other solid, liquid and semi-solid dosage forms for transmucosal administration.

Glycidol methacrylate is a chemically reactive molecule that can potentially be used for introducing unsaturated functional groups to a variety of hydroxyl-containing water-soluble polymers to enhance their mucoadhesive properties. Modification of these polymers with glycidol methacrylate may offer some advantages compared to the use of methacrylic anhydride as a reagent for derivatisation. Water-soluble polymers modified with glycidol methacrylate may exhibit better hydrophilic properties because of the possibility of reaction via epoxide ring opening.

## Figures and Tables

**Table 1:** Details on HECGMA synthesis.

ID	Molar Ratio [HEC]:[GMA]	GMA (μL)	TEA (μL)
HECGMA Low	[1]:[1]	225	240
HECGMA Medium	[1]:[3]	675	240
HECGMA High	[1]:[6]	1350	240

## Data Availability

Not applicable.

## Supplementary Materials

The following supporting information can be downloaded at: https://www.mdpi.com/article/10.3390/polym15010093/s1; Table S1: Standard calibration data of methacrylic acid.; Figure S1: Standard calibration curve of methacrylic acid; Table S2: Calculation of amount of methacrylic acid in HECGMA samples.
