# Supplementary material for: Synthesis of Methacryloylated Hydroxyethylcellulose and Development of Mucoadhesive Wafers for Buccal Drug Delivery
Source: Polymers (Basel). 2022 Dec 26;15(1):93. doi: 10.3390/polym15010093 (PMC9823848; doi:10.3390/polym15010093)
Supplement: Supplementary file 1 [file polymers-15-00093-s001.zip › polymers-2106238-supplementary.pdf]

Supporting Information

Synthesis of methacryloylated hydroxyethylcellulose and development of mucoadhesive wafers for buccal drug delivery

Fhataheya Buang [1,3], Afroditi Chatzifragkou[2], Mohd Cairul Iqbal Mohd Amin[3] and Vitaliy V. Khutoryanskiy[1,*]

[1]Reading School of Pharmacy, University of Reading, Whiteknights, PO Box 224, Reading, RG6 6AD Berkshire, United Kingdom

[2]Department of Food and Nutritional Sciences, University of Reading, Whiteknights, Reading, Berkshire RG6 6AD, Berkshire, United Kingdom.

[3]Centre for Drug Delivery Technology, Faculty of Pharmacy, Universiti Kebangsaan Malaysia, Jalan Raja Muda Abdul Aziz, 50300 Kuala Lumpur, Malaysia.

*Correspondence author: v.khutoryanskiy@reading.ac.uk

Reading School of Pharmacy, University of Reading, Whiteknights, PO Box 224, Reading RG6 6AD, United Kingdom

Table S1. Standard calibration data of methacrylic acid.

No	ID	Concentration (μmol/mL)	Peak area (DAD @ 200 nm)
1	MA 1	59.0	6672.8
2	MA 2	29.5	3462.7
3	MA 3	14.7	1765.2
4	MA 4	7.4	881.8
5	MA 5	3.7	352.9
6	MA 6	1.8	166.8
7	MA 7	0.9	99.9
8	MA 8	0.5	50.2
9	MA 9	0.2	24.6
10	MA 10	0.1	17.3

Figure S1. Standard calibration curve of methacrylic acid

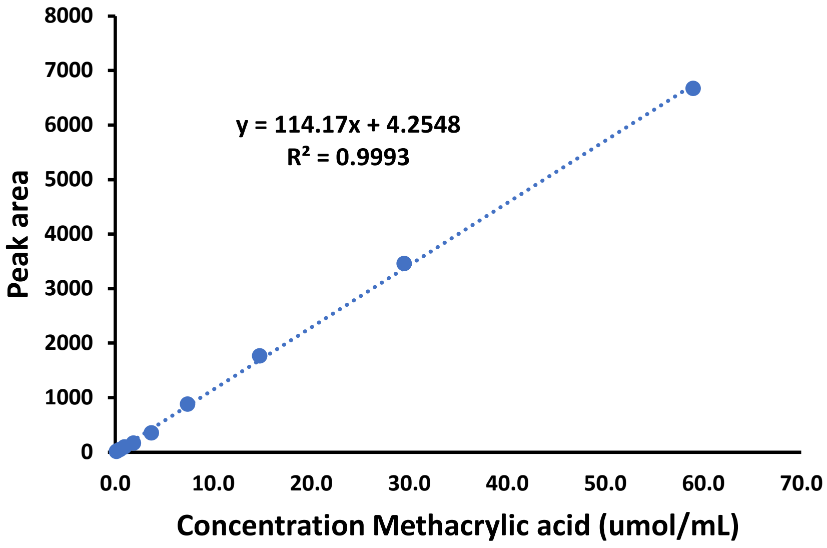

Table S2: Calculation of amount of methacrylic acid in HECGMA samples

Sample	Absorbance	Concentration of methacrylic acid (μmol/mL)	Actual amount of methacrylic acid (μmol/g)
HEC	0.00	0.00	0.00
HECGMA Low	41.10 ± 0.08	0.32 ± 0.03	64.49 ± 5.98
HECGMA Medium	45.60 ± 7.80	0.36 ± 0.03	72.43 ± 6.16
HECGMA High	103.30 ± 23.00	0.87 ± 0.16	173.50 ± 32.84
